# Effect of parasocial relationship on tourist’s destination attitude and visit intention

**DOI:** 10.1371/journal.pone.0265259

**Published:** 2022-04-06

**Authors:** Xiang Zheng, Jian Ming Luo, Ziye Shang

**Affiliations:** Faculty of International Tourism and Management, City University of Macau, Macau, China; Universita degli Studi di Perugia, ITALY

## Abstract

**Background:**

Along with the rapid development of the Internet, the form of destination marketing is becoming increasingly innovative and diverse. Celebrity endorsement via social media, as an effective marketing strategy, has been widely used by various tourism companies to attract and engage with their customers. Previous studies have investigated the various aspects of such endorsement (e.g. the effect of endorser’s attributes) in different contexts (e.g. Hotel, Restaurant, Airline). However, little research has focused on the influence of the tourists’ parasocial relationship with endorsers on destination marketing. Accordingly, the current study aims to explore the role of parasocial relationships on tourists’ destination attitude and visit intention.

**Method:**

This study adopted quantitative method and conducted Partial Least Square-Structural Equation Modelling (PLS-SEM) for data analysis. 498 valid questionnaires were collected from Weibo (One of China’s most popuar social media platforms).

**Results:**

Results suggested that endorser’s credibility positively influenced follower’s parasocial relationship (p<0.001), which in turn positively influenced destination attitude and visit intention (p<0.001). Additionally, the parasocial relationship significantly mediated the relationship between credibility and destination attitude as well as visit intention (p<0.001).

**Conclusion:**

The emerging trend of celerbity endorsement via live-streaming makes tourism destination marketing more diverse and even effective. Through exploring the underlying mechanism of celebrity endorsement, this study can provide destination marketers with insights about how to build and strengthen the tourist’s attitude and visit intention towards destiantion by developing their parasocial relationships with endorsers. This study also adds to the literature of using Partial Least Square-Structural Equation Modelling (PLS-SEM) in tourism and hospiatlity research.

## 1. Introduction

According to the China Internet Network Information Centre [1], the Internet penetration rate has reached 70.4% in China, with nearly 1 billion netizens. Aiming to take advantages of the Internet development, many tourism companies and destination management organisations started using various tools including website, social media and online influencer strategy to help with destination marketing. Ctrip, for example, as China’s largest online travel agent [2], reported that the gross merchandise volume (GMV) from live-streaming has exceeded 2.4 billion Yuan by the third quarter of 2020, attracting more than 170 million views [1]. Interestingly, the chief executive officer of Ctrip also actively participated in such activities through live-streamed videos of himself, which transformed him into an Internet star and turned out to be very effective among customers [3]. Clearly, celebrity endorsement via social media platforms has become an emerging marketing strategy in the tourism field.

Recent trends in celebrity endorsement have led to a proliferation of studies around the theme of the impact of destination endorsers on tourist visit intention, destination brand love and destination attitude [4–9]. Previous research suggests that the potential benefits of adopting celebrity endorsement strategy include stronger emotional bonds between customers and brands [10, 11], product involvement [12], corporate credibility and loyalty [13] and visit intention and destination attitude [5]. Existing research in the early stage also recognises the critical role played by different attributes of endorsers in explaining the influence of celebrity endorsement [14, 15]. Moreover, one variable from the media field, namely, parasocial relationship, has seen its popularity in tourism research in the past few years. Parasoical relationship is a concept that describes an illusory intimacy developed between the media person and the viewers [16]. Previous studies examined the role of parasocial relationship in brand identification and customer citizenship behaviour [17], perceived well-being and visit intention in the context of TV-induced tourism [18], destination brand love [9], perceived brand quality, brand affect and brand preference [19].

Nevertheless, few studies have investigated the role of parasocial relationship with endorsers in destination marketing. This study adapted a three-dimensional credibility model originally proposed by [15], containing attractiveness, trustworthiness and expertise components, to explore the underlying mechanism of celebrity endorsement in tourism destination marketing by applying theories from various fields, such as uncertainty reduction theory, affect transfer theory and social exchange theory into the tourism setting. More specifically, we examine the emerging role of parasocial relationship within the perspective of destination marketing and verify the relationships among attractiveness, trustworthiness, expertise, parasocial relationships, destination attitude and visit intention. We selected Tenzin, one recent star of destination ambassador in China, as representative in the field of celebtity endorsement. This study contributes to our knowledge of the influence mechanism of credibility and the role of parasocial relationship on destination marketing, providing valuable insights to tourism companies and destination management organisations for more effective practices in the future.

The remainder of this paper is organised as follows. Section two gives the literature review of relevant concepts used in this study and section three develops the hypotheses between different constructs. Section four describes the methodology of our study, including endorser selection criteria, measurement scales and data collection and analysis process. Section five presents the main results. Section six discusses on the basis of the key results and concludes the entire study. Section seven details both theoretical and practical implication of the study. Section eight outlines the study’s limitations and proposes further research areas.

## 2. Literature review

### 2.1 Celebrity endorser in destination marketing

Celebrity endorser refers to ‘the individual who enjoys public recognition and who uses this recognition on behalf of a consumer good by appearing with it in an advertisement’ [20] (p. 310). In the past, it broadly referred to movie stars, athletics, politicians, business men, artists and military personnel [20]. Then, with the rapid development of technology, those people famous on social media could also be regarded as ‘Internet celebrities’ as they enjoy wide social presence, which in turn has a strong impact on the audience’s behavior [7, 21]. [20] identified endorsements in four distinct types, namely, the explicit mode (‘I endorse this product’), the implicit mode (‘I use this product’), the imperative mode (‘You should use this product’) and the copresent mode (i.e., in which the celebrity merely appears with the product). Celebrity endorsement has received remarkable attention from many scholars, and previous studies found that it has a positive effect on brand awareness and loyalty [22], attitudes towards advertising messages [23], brand attitudes [24], brand awareness [25] and purchase intention [26–30]. In the tourism field, research on the impact of celebrity endorsers on destination marketing concentrates on two perspectives. The first perspective focuses on different attributes of celebrity endorsers. Van der Veen and Song [6] suggested that endorser’s nativity could influence the endorsement effectiveness. Glover [31] pointed out the potential effects of celebrity image on destination awareness and purchase decision. The second perspective focuses on different characteristics of tourists. Magnimi et al. [32] revealed how different audiences with different demographic characteristics respond differently to celebrity endorsers. Parents in particular pay extra attention to matching images. Female and young audiences exhibit active responses to a celebrity’s physical attractiveness, whereas older customers rely more on a celebrity’s trustworthiness. McCartney and Pinto [33] found that when it comes to travel decisions, those travelers who are younger and earning middle income tend to be more easily affected by celebrity endorsers. In addition, many studies looked into the role of celebrity involvement in destination image and visit intention [34, 35].

Although celebrity endorsers received abundant attention from researchers, little study has explored its role in destination marketing from the perspective of parasocial relationships. Zhang et al. [9] explored how the credibility of endorser and tourist’s parasocial relationships influence tourist destination brand love. Su et al. [36] examined the effect of parasocial relationship between customer and TV character on people’s attitude towards a destination.

### 2.2 Endorser credibility

Credibility is often explained as the good qualities of the communicator that may affect the receiver’s acceptance of the related message [15]. In the context of marketing, it reflects the degree to which the information source is recognised as an expert on the issues of discussion and can be credited to offer objective opinions on the subject [15]. By incorporating both the source-credibility model by [37] and the attractiveness model by [38], three dimensions of celebrity endorser’s credibility were identified and tested as reliable and valid by [15], namely, attractiveness, trustworthiness and expertise. Physical attractiveness refers ‘to the degree to which one’s facial image elicits favourable reactions from others’ [39] (p.47). Joseph [40] also concluded that attractive communicators tend to be liked more and can positively influence the product they are endorsing. In other words, enhancing communicator’s attractiveness could lead to a positive change in customer attitude [41, 42]. Trustworthiness is defined as the perceived desire of the information source to provide valid assurance, whilst expertise is defined as the perceived capacity of the information source to provide valid assurance [20]. Previous studies demonstrated that customers’ attitude and behaviour towards a certain brand could be influenced by the perceived credibility of brand endorsers [26, 43]. In addition, Ohanian [15] summarised after reviewing many credibility literatures that sources in high credibility are more persuasive than sources in low credibility and that highly credible sources lead to more obedience in behaviour than do less credible sources.

Many studies have examined the function of endorser credibility in destination marketing in the tourism industry. Van deer Veen and Song [6] found that endorser’s attractiveness positively affects audience’s attitude towards the advertisement and destination, thus indirectly influencing the visit intention. Zhang et al. [9] suggested that the perceived trustworthiness and expertise of a celebrity endorser might have a positive impact on tourists’ brand love towards a destination via parasocial interaction. Kim et al. [44] pointed out that expertise, as one dimension of endorsers’ credibility, is found to be the most relevant factor that associates with brand equity and destination attachment in the context of festival. ven der Veen [11] argued the importance of a ‘right fit’ between the endorser and the destination, and Glover [31] further advocated a three-way match among the destination image, celebrity image and the tourist’s self-image to boost the efficacy of celebrity endorsements of destinations.

### 2.3 Parasocial relationships

The concept of parasocial relationships is derived from and closely related to the concept of parasocial interactions, which is originally put forward by [16]. They described parasocial relationship as the enduring and illusionary intimacy that the media audience could develop through repetitive interactions whether verbally or bodily with the media persona. The main observation of this theory was some social and communicative behaviours such as greet, wink, gaze and direct communication acts from media performers could induce responses from viewers as if they would in real-life interactions [16]. At the early stage, this theory was used widely in the context of various mass media platforms―radio, television and the movies. Levy [45] found that people who established a parasocial relationship with newscasters (one typical example of popular mass media performers at that period) through more interactions watched news programmes more frequently. Rubin and Step [46] examined the role of parasocial relationship on radio listening frequency. Looking forward to the age of new media, the parasocial relationship theory apparently started to be adopted in the context of various social media personas, such as Twitter celebrities [47], Instagram and YouTube bloggers [48, 49]. Previous studies showed that parasocial relationship between viewers and performers could positively influence purchase intention [49], social commerce intention [50], brand loyalty as well as the willingness to share further information of the brand [51].

In the field of tourism, parasocial relationship also received adequate attention from many scholars. From the viewpoint of tourism product brand, Lee and Lee [52] investigated the relationship between customer’s PSR with hotel brand and their self-brand connection and brand usage intention. Likewise, the influence of customer’s PSR with hotel company on their continuous intention of using branded app was also examined [53]. Moreover, the relationship among PSR, brand identification and customer citizenship behaviour were identified in previous study [17]. From the viewpoint of tourism destination marketing, Kim and Kim [54] examined how the feelings of intimacy and/or the close bond that audience developed with the drama performers affected audience’s behavioural intention in film tourism. Choi et al. [55] tested the role of PSR on travel satisfaction and community satisfaction, whilst Zhang et al. [9] examined the role of PSR on tourist destination brand love and Shang and Luo [56] studied the relationship between PSR and place attachment. Su et al. [36] found that if highly perceived cultural proximity exists, the viewer’s PSR with and attitude towards the character are associated with viewer’s attitude towards the destination. The relationship among young audiences’ PSR, perceived well-being and travel intentions were also examined by the new study [18].

### 2.4 Tourist attitude towards destination

According to the theory of planned behaviour (TPB), attitude towards a behaviour was defined as ‘the degree to which a person has a favourable or unfavourable evaluation or appraisal of the behaviour in question’ [57] (p.188). Individuals with a more favourable attitude about the behaviour have a greater desire to engage in the behavior [58]. In the tourism setting, destination attitude refers to predispositions or feelings of tourists towards a vacation place based on various perceived attributes of products and service in that destination [59]. In addition, tourist attitude consists of three dimensions, namely, cognitive, affective and behavioural components [60]. For destination marketing, Um and Crompton [61] found that tourist attitude has a strong impact on selecting potential travel destination and deciding the final destination. Jalilvand et al. [62] indicated that tourist attitude has a strong relationship with travel intention. Souiden et al. [63] examined the relationship among destination personality, tourist attitude and behavioural intention. Similarly, Lee [64] revealed that future tourist behaviour is also affected by tourist attitude.

### 2.5 Tourist visit intention

On the notion of planned behaviour, intention relates to how ready individuals are to attempt and how much effort they are willing to put in with the aim of acting out the behaviour [57]. In the context of tourism, it refers to the tendency of potential tourists to visit a specific destination, which is triggered by different information from both internal and external sources [65]. Lu et al. [66] revealed that those people with stronger intention to visit a destination will more likely to do so eventually. In tourism field, many previous study examined the factors that influence visit intention such as hedonism and tourist experience [67], generativity, experience expectation, and motivation [68], and satisfaction [69].

## 3. Hypothesis development

### 3.1 Endorser’s credibility and parasocial relationship

In the human communication field, uncertainty reduction theory (URT) is often used to illustrate the communication process between strangers [70]. This theory was originally put forward by [71] who indicated that we tend to experience a certain degree of uncertainty when interacting with others due to insufficient information and a number of possible alternatives. However, the uncertainty level is relatively high at the initial stage and tends to decrease as time goes, especially when people attempt to reduce the uncertainty through various means. The main objective of uncertainty reduction is to make sense of things, which usually involves the increasing ability to explain or predict self and others’ behaviour [70]. According to [71], seven components related to uncertainty at the beginning stage of communication were identified, including the amount of verbal communication, nonverbal affiliative expressiveness, intimacy level and so on. For this study, as we introduced earlier, endorser’s credibility is a term that refers to the level to which the media person could provide reliable and sufficient information about the relevant topic to audiences, and the PSR means the imaged intimacy that developed in the communication process. Therefore, under the framework of URT, the increase in endorser’s credibility may lead to an increase in PSR between endorsers and audiences.

Other than theoretical foundation, previous studies also give empirical support to these two variables. Chung and Cho [72] found the mediated role of PSR between social media interaction and source credibility in the context of Korean Wave fans. Reinikainen et al. [73] pointed out that the audience’s PSR with online influencer and the influencer’s perceived credibility are positively related. Similarly, source credibility was found to positively influence follower’s PSR with influencers [74]. Hence, based on both theoretical and empirical foundations, the following hypotheses were developed:

**H1:** Destination endorser’s attractiveness positively influences viewer’s parasocial relationship with endorser.
**H2:** Destination endorser’s trustworthiness positively influences viewer’s parasocial relationship with endorser.
**H3:** Destination endorser’s expertise positively influences viewer’s parasocial relationship with endorser.

### 3.2 Parasocial relationship and destination attitude

Previous studies used affect transfer theory (ATT) to examine the role of affect in different contexts, including customer’s judgement of a product [75], advertising evaluation [76] and celebrity endorsement [77]. The ATT was initially put forward by [78], suggesting that people’s affect towards one object would transfer to another if new items could match the existing one. When applying ATT into celebrity endorsement [77], found that customer’s affect towards brand spokesperson could be transferred to the endorsing brand when the two were congruent. In addition, higher brand affect from customers was found when a match up existed between the spokesperson and the brand itself. For the current study, audience PSR with the destination endorser is explained as an intimacy, which in other words, an affect that the audience have towards the endorser. Therefore, we assume that customer’s affect towards the endorser could be transferred to the endorsed destination, provided they can match up with each other. Empirically [9], revealed the positive effect of PSR on the destination brand love of both past and future tourists, under celebrity endorsement situations. Therefore, the following hypothesis was developed:

**H4:** The viewer’s parasocial relationship with the endorser positively influences viewer’s destination attitude.

### 3.3 Parasocial relationship and visit intention

As proposed by [79] (p.13), social behaviour is regarded ‘as an exchange of activity, tangible or intangible, and more or less rewarding or costly, between at least two persons’. This social exchange theory (SET) admits that human’s behaviour can be regarded as the outcome of cost-benefit analysis when the interaction between two people happens. Burns [80] further stated that the rewards we are expected to gain drive us to interact with others, whilst others also gain benefits from us. Consequently, an interdependent relationship may be created by doing so. SET has been modified and improved over time and has been applied to various fields such as interpersonal relationships and human powers, among others. For the present study, PSR is also a kind of relationship that audiences developed towards endorsers. One the one hand, the reward that the audience obtain from developing PSR with endorsers is probably the feeling of intimacy although it is sometimes one-sided and self-imaged. On the other hand, endorsers wish audience could pay to visit that specific destination they are promoting, which could also be seen as a reward from audiences. Under the framework of SET, humans tend to give something (cost) in exchange of a valued reward. Therefore, we assume that audiences will have a strong intention to visit the endorsed destination as a form of exchange behaviour.

In addition, previous studies empirically tested the relationship between PSR and visit intention. Research conducted by [81] showed the mediating role of PSR between source credibility and visit intention. Bi et al. [18]’s recent study found the significant indirect impact of PSR on young people’s TV-induced travel intentions through perceived well-being. Thus, the following hypothesis was developed:

**H5:** The viewer’s parasocial relationship with the endorser positively influences viewer’s visit intention.

### 3.4 Destination attitude and visit intention

**Fig 1:**
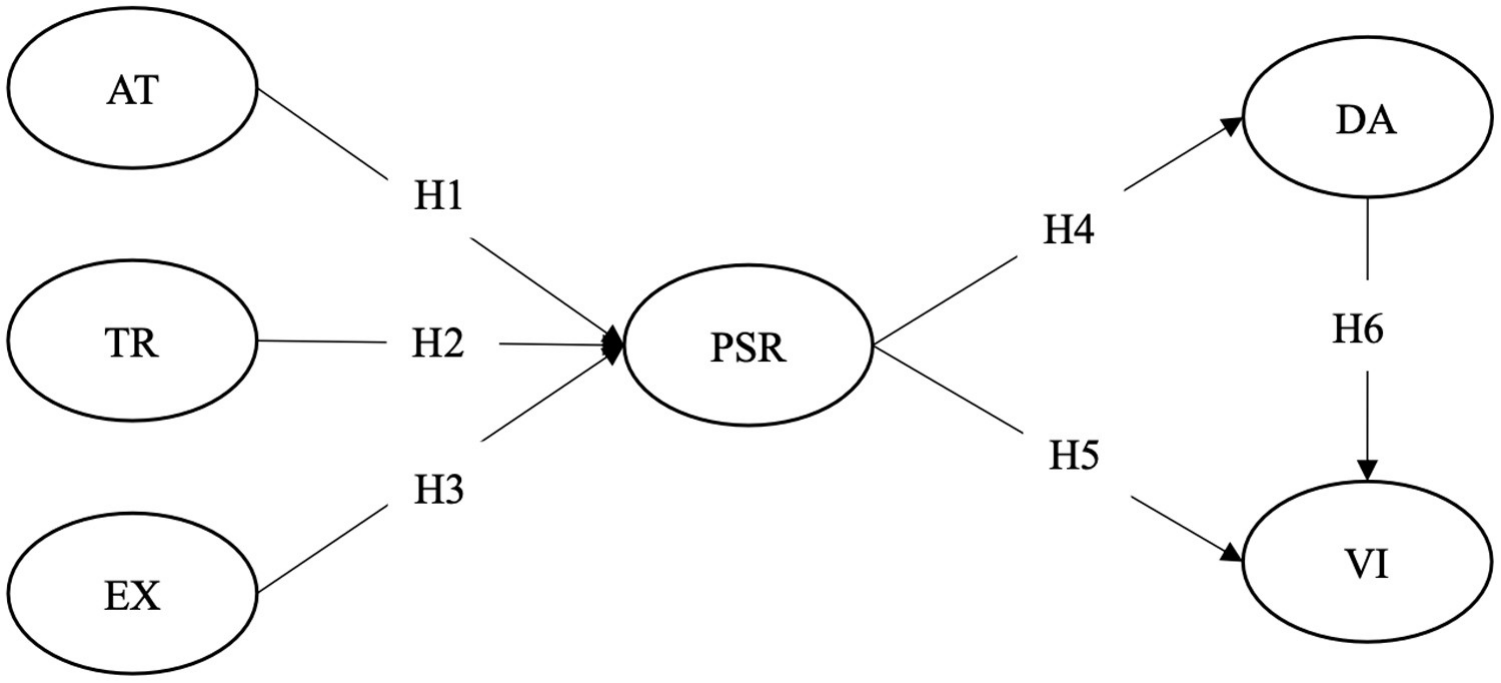
Proposed research model.

As the concept of planned behaviour suggests, people tend to act in line with their intentions, whilst their intentions to perform a behaviour is influenced by their attitude towards that behaviour. In other words, people’s attitude has a strong power in predicting and understanding their actions [58]. Therefore, if we apply that theory to the tourism context, tourists’ intention to visit a certain destination might be interpreted to be influenced by their attitude towards that destination. Many studies in fact gave empirical support to the relationship between tourist’s destination attitude and visit intention. Loureiro [82] revealed the influence of favourable tourist attitude on destination visit intention. In [81]’s study, Chinese tourists’ intention to visit Australia was found to be positively influenced by their attitude towards that destination. A similar result also appeared in [83]’s research examining the relationship among e-Word-of-Mouth, tourists’ attitude and visit intention to Jordan. Doosit et al. [84] verified the same result in their study. Therefore, the following hypothesis was developed. Fig 1 shows our research model.

**H6:** The viewer’s destination attitude positively influences viewer’s visit intention to that destination.

## 4. Methodology

### 4.1 Selection of destination endorser

For this study, we selected Tenzin, a young Tibetan, as the target destination endorser. Our choice is largely based on the popularity of Tenzin and its resulting effects in tourism businesses. In November 2020, Tenzin became a phenomenal social media star after photos of his pure eyes and smiles shown in a 10-sec video have gone viral online. His official account in Weibo (Chinese most popular social media platform) received nearly 1.5 million followers at the time we began conducting our study. Then, he was appointed official destination ambassador for Litang County, Sichuan Province, where he was born and living [85]. In addition, Tenzin gained widespread publicity from various media outlets both domestic (e.g. China Xinhua News) and overseas (e.g. Forbes). Driven by his overwhelming popularity, Tenzin made the first live-streaming show online, which attracted more than 100 thousand viewers and received more than 1 million likes only within 1 hour [86]. In the business world, the results are also eye-catching. According to the data released by Ctrip (the largest online travel agent in China), the search volume for destination Litang increased noticeably by 620 percent during the time period between November 20 to November 30 in 2020 [87]. Users who searched that destination are not only within Sichuan Province but also from other parts of China. Similarly, Qunar (another popular OTA in China) reported that till November 25, the hotel booking volume in the Ganzi area, where Litang is located, increased by 89 percent from the last year. To sum up, Tenzin is clearly a recent hot and effective destination endorser who attracts much attention from the public. Therefore, we thought Tenzin is a sufficient representative to be selected as destination endorser in this study.

### 4.2 Measurement and data collection

This study used previously developed measuring scales and designed self-administrated questionnaire. Minor adjustments were made to reflect the context of tourism destination marketing. A pilot test was conducted on 30 students majoring in tourism and hospitality management and according to their feedback, we revised the English-Chinese word translation for better understanding. Moreover, four screening questions were developed to ensure that the respondents not only know Tenzin and his identity as a Litang destination endorser but also to guarantee that they are non-local residents of Litang who have not been to that place before. The screening questions are 1) Do you know Tenzin? (with Tenzin’s image attached); 2) Do you know Tenzin as a destination endorser of Litang? 3) Are you a local resident of Litang? 4) Have you been to Litang? All the scales adopted in this study have been proven reliable and valid by previous research. All items were rated on a 5-point Likert Scale ranging from 1 (strongly disagree) to 5 (strongly agree). In sum, 26 measurement items for six constructs are included in the questionnaire. Scales for each construct are as follows:

Credibility was measured by attractiveness, trustworthiness and expertise components; each of them was measured by 4 distinguishing items [15].

Parasocial relationship was measured with 8 items [51]; [88–90].

Destination attitude was measured with 3 items adapted from previous studies [91, 92].

Visit intention was measured with 3 items from [93].

For data collection, convenience sampling method was used by distributing online questionnaire in chat groups related to Sichuan Tourism in Weibo. Given our assumption that followers in those channels are more easily to gain information about the Sichuan Tourism, they might be more familiar with the least news regarding Sichuan tourism (which includes Litang destination) than other Weibo users. The number of post was more than 174 thousand in that channel, with nearly 250 million views. Thus, distributing our questionnaire there is reasonable. In total, 746 questionnaires were collected within 1 month. Then, we carefully evaluated each response and eliminated invalid samples. Our selection processes mainly constitute two aspects. On the one hand, in the first screening questions part, those respondents who didn’t know Tenzin or didn’t know Tenzin as a destination endorser of Litang county or the respondents are the local people of Litang county or they have been to Litang county before were regarded as invalid responses. Because the target population of this research is those people who not only can recognise Tenzin and know his identity as destinstion endorser of Litang county but also are not the local people who have not been to Litang before. On the other hand, those responses which had the same scores in each 5-point Likert Scale question of measurement items were also counted as invalid [94]. Finally, 498 valid responses were retained.

## 5. Results

SPSS was employed to conduct a demographic statistical analysis, and then SmartPLS 3.3 software was adopted to estimate a structural equation model. SmartPLS 3 is a milestone in latent variable modeling, combining state of the art methods (e.g. PLS-POS, IPMA, complex bootstrapping routines) with an easy to use and intuitive graphical user interface, which is widely adopted in tourism and other fields [17, 95–99].

### 5.1 Respondent profile

Table 1 describes the statistics about the demographic profile of respondents. Among the sample, 67.6% are female participants. Majority of the respondents have a bachelor’s degree and are aged between 18 to 35. Nearly 80% of the participants are working or studying.

**Table 1:** The demographic profile of respondents.

Variable		Frequency (n = 498)	%
Gender	Female	337	67.6
	Male	161	32.4
Marital Status	Married	158	31.7
	Other	340	68.3
Age	18–25	161	32.4
	26–35	198	39.7
	36–45	117	23.5
	46–55	15	3.1
	56 or above	7	1.3
Education	High school or below	76	15.2
	Undergraduates	337	67.6
	Graduates or above	85	17.2
Occupation	Working	178	35.7
	Student	211	42.4
	Housewife	16	3.2
	Retired	5	1.1
	Others	88	17.6

### 5.2 Measurement model

We used statistical software SmartPLS to conduct confirmatory factor analysis (CFA). The reliability, convergent validity and discriminant validity of all scales were satisfactory. Table 2 presents the summary of CFA results for the measurement model which includes all scale items. Both values of composite reliability (CR) and Cronbach’s alpha are above the recommended level 0.7 (ranging from 0.935 to 0.968 and from 0.896 to 0.957), indicating a satisficatory reliability of all measurement scales. Then, all the factor loadings are above 0.7 (ranging from 0.791 to 0.949), and all the average variance extracted (AVE) values are greater than the threshold 0.5 (ranging from 0.738 to 0.885), showing well accepted convergent validity among all measuring constructs [100]. Data normality, which is indicated by kurtosis and skewness ranging from -1 to 1, is also suitable. Finally, the discriminant validity is achieved by obtaining the greater value of the square root of each AVE than its construct correlations [101], indicating well statistical distinction among constructs (see Table 3). However, for PLS path modelling, we would like to make our readers aware that the global goodness-of-fit index is lacking since PLS-SEM focuses on the discrepancy between the observed (in the case of manifest variables) or approximated (in the case of latent variables) values of the dependent variables and the values predicted by the model in question [102]. Many scholars who adopted PLS modelling have pointed out the lack of an index that can provide researchers with a global validation of the model [99, 103].

**Table 2:** Summary for confirmatory factor analysis.

Item	Factor Loadings	Cronbach’s Alpha	CR	AVE	Kurtosis	Skewness
***Trustworthiness (TR)***						
TR1: I feel Tenzin was honest	0.931	0.957	0.968	0.885	0.472	-0.754
TR2: I consider Tenzin trustworthy	0.944				0.275	-0.625
TR3: I feel Tenzin was truthful	0.949				0.641	-0.846
TR4: I consider Tenzin earnest	0.939				0.792	-0.964
***Attrativeness (AT)***						
AT1: I consider Tenzin very attractive	0.931	0.932	0.952	0.831	-0.262	-0.397
AT2: I consider Tenzin very stylish	0.896				-0.378	-0.191
AT3: I think Tenzin is good looking	0.883				-0.605	-0.333
AT4: I think Tenzin is sexy	0.936				-0.636	-0.302
***Expertise (EX)***						
EX1: I feel Tenzin knows a lot about the travel destination (i.e., Litang)	0.863	0.926	0.947	0.818	0.179	-0.523
EX2: I feel Tenzin is competent to make assertions about the travel destination	0.924				-0.248	-0.255
EX3: I consider Tenzin an expert on the travel destination	0.913				-0.251	-0.109
EX4: I consider Tenzin sufficiently experienced to make assertions about the travel destination	0.916				-0.406	-0.286
***Parasocial Relationship (PSR)***						
PSR1: I look forward to watching Tenzin on his channel	0.844	0.949	0.957	0.738	-0.529	-0.334
PSR2: If Tenzin appeared on another program, I would watch that video	0.891				-0.81	-0.067
PSR3: When I’m watching Tenzin, I feel as if I am part of his group	0.868				-0.947	0.016
PSR4: I think Tenzin is like an old friend	0.868				-0.732	0.103
PSR5: I would like to meet Tenzin in person	0.869				-0.732	0.143
PSR6: If there were a story about Tenzin in a newspaper or magazine, I would read it	0.856				-0.653	-0.369
PSR7: Tenzin makes me feel comfortable as if I am with friends	0.88				-0.763	-0.167
PSR8: When Tenzin shows me how he feels about the destination, it helps me make up my own mind about the destination	0.791				-0.232	-0.586
***Destination Attitude (DA)***						
DA1: Very bad- very good	0.932	0.896	0.935	0.828	0.207	-0.463
DA2: Very unfavorable–very favorable	0.933				0.00	-0.409
DA3: Very negative–very positive	0.864				0.58	-0.726
***Visit Intention (VI)***						
VI1: I would like to visit this place (i.e., Litang) in the future	0.921	0.901	0.938	0.834	0.582	-0.815
VI2: It is likely that I visit this place in the future	0.921				0.345	-0.686
VI3: I will intend to visit this place in the future	0.897				-0.087	-0.525

**Table 3:** Discriminant validity.

	PSR	AT	DA	EX	TR	VI
PSR	**0.859**					
AT	0.754	**0.912**				
DA	0.499	0.493	**0.91**			
EX	0.608	0.586	0.529	**0.904**		
TR	0.656	0.699	0.595	0.522	**0.94**	
VI	0.535	0.531	0.627	0.497	0.49	**0.913**

### 5.3 Structural model testing

**Fig 2:**
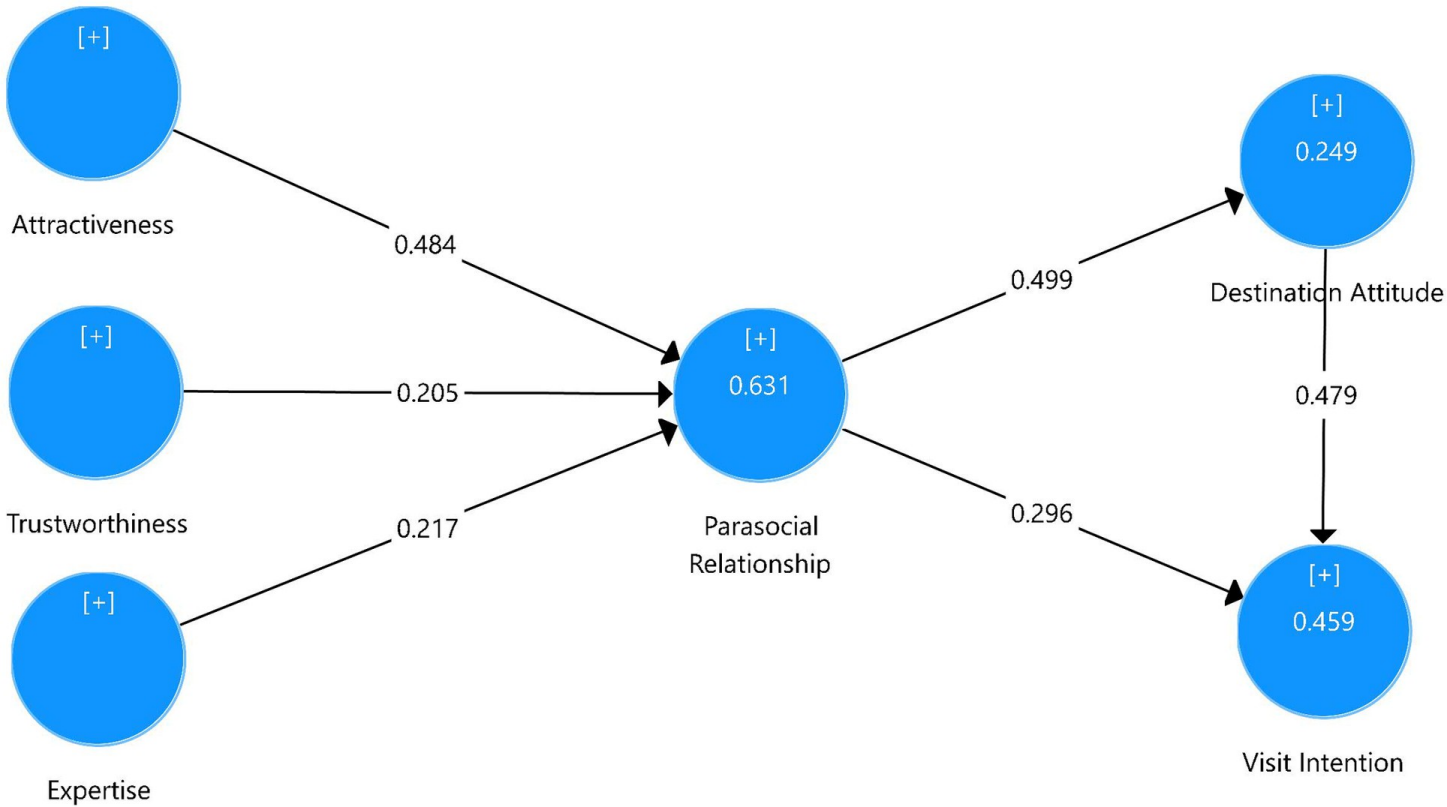
Path Coefficients in the structural model.

The current study adopted the Bootstrap method to test the hypothesis and meditating effect. The bias-corrected bootstrap confidence interval estimation method is also used to conduct interval estimation. The confidence coefficient was set at 95%, and the Bootstrap was set to run 5000 times. As shown in Table 4, destination endorser’s attractiveness, trustworthiness and expertise were strongly associated with parasocial relationship, supporting H1, H2 and H3. Parasocial relationship was significantly related to destination attitude and visit intention. At last, destination attitude was significantly related to visit intention. These three results support H4, H5 and H6, respectively. Destination attitude was also found to mediate the effect of parasocial relationship on visit intention. The R^2^ values related with each construct were satisfactory (R^2^ of Parasocial Relationship = 0.631, R^2^ of Destination Attitude = 0.249, R^2^ of Visit Intention = 0.459), see Fig 2 and Table 5.

**Table 4:** Direct path for the structual model.

Hypothesis	Coefficient	Standard Deviation	T Values	P Values	Confidence intervals bias corrected	
					2.50%	97.50%
AT -> PSR	0.484	0.044	11.095	<0.001	0.399	0.572
DA -> VI	0.479	0.044	10.961	<0.001	0.388	0.558
EX -> PSR	0.217	0.039	5.54	<0.001	0.139	0.291
PSR -> VI	0.296	0.044	6.748	<0.001	0.211	0.382
PSR -> DA	0.499	0.038	13.219	<0.001	0.421	0.569
TR -> PSR	0.205	0.046	4.468	<0.001	0.109	0.291

**Table 5:** Q^2^ and R^2^.

Variables	Q^2^	R^2^
VI	0.376	0.459
DA	0.203	0.249
PSR	0.458	0.631

In short, all hypotheses were supported in the results (see Tables 4 and 6). Moreover, Table 6 shows the indirect impacts of destination endorser’s attractiveness, truthworthiness and expertise on tourists’ visit intention through parasocial relationship, and destination attitudes were significant (p < 0.001). In addition, the indirect impact of parasocial relationship on tourists’ visit intention through destination attitude was also significant (p < 0.001). To sum up, both direct and indirect impacts of the above constructs on tourists’ destination attitude and visit intention were all significant in this examined research model.

**Table 6:** Mediation tests results.

Hypothesis	Coefficient	Standard Deviation	T Values	P Values	Confidence intervals bias corrected	
					2.50%	97.50%
EX -> PSR -> VI	0.064	0.016	4.032	<0.001	0.037	0.098
TR -> PSR -> VI	0.061	0.016	3.728	<0.001	0.032	0.096
AT -> PSR -> DA	0.242	0.025	9.651	<0.001	0.195	0.293
TR -> PSR -> DA	0.102	0.026	3.858	<0.001	0.051	0.155
AT -> PSR -> DA -> VI	0.116	0.016	7.397	<0.001	0.088	0.149
PSR -> DA -> VI	0.239	0.025	9.408	<0.001	0.191	0.291
EX -> PSR -> DA -> VI	0.052	0.011	4.74	<0.001	0.032	0.075
TR -> PSR -> DA -> VI	0.049	0.013	3.863	<0.001	0.026	0.076
EX -> PSR -> DA	0.108	0.022	4.895	<0.001	0.066	0.152
AT -> PSR -> VI	0.143	0.025	5.709	<0.001	0.098	0.195

## 6. Discussion and conclusion

Based on uncertainty reduction theory, affect transfer theory and social exchange theory, the current study explains the underlying mechanism of how destination endorser affects tourist attitude and visit intention towards that place. Firstly, the results support uncertainty reduction theory by revealing that endorsers’ credibility (attractiveness, trustworthiness, expertise) can positively influence the parasocial relationship between audience and endorser. Audiences who perceived endorsers at a high level of attractiveness, trustworthiness and expertise are more likely to develop an intimate relationship with them. For our study, it is likely that Tenzin’s warm and pure smile and his expertise of Litang reduce viewers’ uncertainty about him and start to regard him as an attractive, reliable and professional destination endorser. Consequently, a parasocial relationship between viewers and Tenzin is established. Indeed, this outcome is consistent with prior findings obtained by [73, 74] who also found that influencers’ credibility is positively related to viewers’ parasocial relationship with them. Our study verified that outcome in the context of tourism destination endorsement.

Secondly, the results support affect transfer theory by suggesting that audiences’ parasocial relationship with endorsers can positively influence audiences’ attitude towards the endorsed destination. This finding can be well explained by affect transfer theory because our study pointed out that audiences’ favourable experiences derived from the established parasocial relationship with Tenzin in this study could be directly transferred to the attitude towards the specific destination, namely, Litang County. This finding is consistent with that of [77] who found that audiences’ emotion towards a brand spokesperson could be transferred to the endorsed brand. Ye et al. [17] also obtained a similar result that customers’ parasocial relationship with companies’ spokesperson could influence brand identification, supporting the meaning transfer model. Our study contributes to the literature and makes one of the earliest attempts to examine the role of parasocial relationship with endorsers in destination marketing.

Thirdly, the present study points out that audiences’ parasocial relationship with endorsers is positively associated with audiences’ visit intention towards the endorsed destination. This outcome can find solid foundation in social exchange theory, which proposed that exchange behaviour would occur when people were awarded with something valuable and therefore tended to give something in return. In our study, we could conclude that followers’ positive relationship with Tenzin would lead to exchange behaviour, inducing among the audience a strong intention to visit the destination that Tenzin was representing. Finally, the parasocial relationship significantly mediates the relationship between credibility and destination attitude as well as visit intention. Therefore, the results helped us answer the research questions mentioned above and uncover this relatively less-focused but important area.

In conclusion, with the flourishing of new technologies in recent years, tourism companies and destination management organisations try their best to take advantages of the new things. Among them, celebrity endorsement receives plenty of attention. However, the underlying mechanism of using celebrity endorsers to promote the destination is relatively unknown. The study introduced the concept of parasocial relationship to illustrate how it can influence tourist’s destination attitude and visit intention. The source credibility model was also verified in the current study. To summarise, the main results of this research are as follows: 1) endorsers’ attractiveness, trustworthiness and expertise have a positive influence on followers’ parasocial relationships, which in turn positively influence destination attitude and visit intention, and 2) the parasocial relationship significantly mediates the relationship between credibility and destination attitude as well as visit intention. These results highlight the importance of selecting credible endorsers and building parasocial relationships in destination marketing.

## 7. Theoretical and practical implication

Theoretically, this work makes several significant contributions to academia. Firstly, we adopted the source credibity model to verity the relationship among attractiveness, trustworthiness, expertise, parasocial relationships, destination attitude and visit intention, giving empirical support to the existing literature [72–74, 81, 82, 104]. Secondly, although previous literatures showed that endorsers in destination marketing received considerable amount of academic attention, literature about the influence mechanism of endorsers’ credibility on tourist visit intention and destination attitude remains extremely limited. This study applies theories from various fields such as the human communication and psychology fields to the social science area, with an attempt to understand the role of credibility and parasocial relationship in tourism destination marketing. From the viewpoint of uncertainty reduction theory, credible endorsers can foster audiences’ parasocial relationship with them. From affect transfer theory, audiences’ positive experience elicited from parasocial relationship can be transferred to the endorser destination. From social exchange theory, positive relationship between audience and endorsers can lead to exchange behaviour, namely, show intention to visit the destination. Borrowing classic theories from other fields and applying them to tourism setting show the beauty of interdisciplinary knowledge. In addition, this research adds to the literature of using PLS-SEM in the hospitality and tourism research.

Practically, this study has the following contributions to business practice. Firstly, tourism companies and DMOs could consider using credible endorsers to increase the effectiveness of their marketing campaign. Many tourism companies nowadays tend to promote their products by releasing information about price and product characteristics (e.g. travel routines). However, this kind of information is usually regarded as too purpose-oriented and even serious, lacking reliability and appetency. According to our study, hiring credible endorsers to promote the destination is likely to be more effective if parasocial relationship could be established between endorsers and audiences. Therefore, companies should consider this strategy. Moreover, evaluation of endorsers could be based on three main criteria, namely, attractiveness, trustworthiness and expertise. Secondly, for marketing campaign activities, practitioners should try to design undertakings that could help build positive relationships between endorsers and followers. One way to accomplish this goal, according to our study, is to foster parasocial relationship between the two. When conducting online endorsement, endorsers could design sections that include ice-breakers to reduce the uncertainty that audience have towards endorsers, which in turn could help establish parasocial relationships. For instance, a casual Q&A session between the audience and the endorser could be developed to acquaint the two parties with each other at the initial stage and create an easy-going overall atmosphere. In this way, endorsers are likely to establish an emotional connection with audiences and therefore strengthen the parasocial relationships. Thirdly, this study suggested that audiences’ positive experience obtained from parasocial relationship could be transferred to their destination attitude and consequently affect their visit intention. In other words, for those audiences who thoroughly enjoy such an experience will be stimulated to show buying behaviour regarding the endorsed products and services in return. Thus, tourist businesses may use a range of emerging technologies, such as artificial intelligence (AI) and virtual reality (VR), to enhance audiences’ experiences (e.g. immersive and novel experiences), which could ultimately result in increased visit intention.

## 8. Limitations and future research

Several limitations of this study must be acknowledged. Firstly, although we received a relatively large number of survey response from Weibo chat groups, the results cannot be generalised to all Chinese fans of the destination endorser and thus, not even worldwide. Secondly, this research is a cross-sectional study which implies that we collected data during a certain time period and thus may lack representativeness. Thirdly, the soft-modelling approach (i.e. PLS-SEM) this study adopted has some limitations. Although this approach requires no normality, the lack of a global goodness-of-fit index is a major shortcoming of this method [95, 99, 102]. Compared with Covariance-based Structural Equation Modelling (CE-SEM), the use of PLS-SEM is still limited despite recent growth in various fields include tourism [95, 96]. Last but not the least, many other variables probably exist, which also influence the role of parasocial relationship on destination attitude and visit intention but have been excluded in the scope of this study.

Future study could focus on the comparative aspects, such as male endorsers vs female endorsers, local endorsers vs nonlocal endorsers, to see whether the results vary. Further information on this field could help us gain a better understanding about the role that parasocial relationship plays in destination marketing.

## Supporting information

S1 Data (CSV)
